# Ultrastructural Description of Amphid Neurons in the Pine Wood Nematode Indicates Concurrent Evolution of the Stylet and Specific Neurons

**DOI:** 10.1002/cne.70114

**Published:** 2025-11-12

**Authors:** Taisuke Ekino, Ryoji Shinya

**Affiliations:** ^1^ School of Agriculture Meiji University Kawasaki Kanagawa Japan; ^2^ Research Center for Global Agromedicine Obihiro University of Agriculture and Veterinary Medicine Obihiro Hokkaido Japan; ^3^ Department of Agro‐environmental Science Obihiro University of Agriculture & Veterinary Medicine Obihiro Hokkaido Japan

**Keywords:** *Caenorhabditis elegans*, *Heterodera*, *Meloidogyne*, plant‐parasitic nematode, sensory ecology, serial section transmission electron microscopy, tactile receptor, umwelt, RRID:WB‐STRAIN:WBStrain00041429, RRID:SCR_003297, RRID:SCR_008606

## Abstract

Understanding how animals perceive environmental stimuli is essential for reconstructing the evolution of their sensory systems. Nematodes provide a useful model for studying sensory adaptation due to their relatively simple nervous systems and broad ecological diversity. The amphid, the primary sensory organ in nematodes, has been well characterized in *Caenorhabditis elegans* and other bacterivorous species. However, comparatively little is known about amphid structures in nematodes with different ecological niches.​ In this study, we performed serial section transmission electron microscopy and three‐dimensional reconstruction of amphid neurons in *Bursaphelenchus xylophilus*, a fungal‐feeding, plant‐parasitic nematode. We identified 13 amphid neurons, five of which showed a distinct morphology and are designated as type V neurons.These neurons were previously described as outer accessory cilia in other stylet‐bearing nematodes, and had not been observed in bacterivorous species. Type V neurons exhibited trifurcated cilia that extended toward each lip and were structurally reminiscent of mechanosensory neurons.​ The presence of type V neurons only in stylet‐bearing nematodes is consistent with the hypothesis that these neurons may have evolved in association with the stylet. Their trifurcated cilia suggest a potential role in detecting mechanical cues during lip contact with substrates, which could trigger stylet ejection. Alternatively, they may also contribute to other sensory modalities. Our findings reveal that fungal‐feeding plant‐parasitic nematodes possess amphid sensory architectures that differ markedly from those of bacterivorous species.

## Graphical abstract

Nematodes possess simple nervous systems yet show broad ecological diversity. In the fungal‐feeding plant‐parasitic nematode *Bursaphelenchus xylophilus*, we identified type V neurons in the amphid. These neurons, likely associated with the stylet, have trifurcated cilia and may have a potential mechanosensory role in triggering stylet‐thrusting behavior against fungal or plant cells.

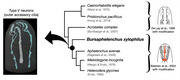

## 1 Introduction

Different animals evolve diverse sensory systems that allow the perception of important cues throughout their life histories. Nematodes are good model organisms for understanding the evolution of the sensory system, for at least two reasons. First, we have comprehensive knowledge of the nervous system of the bacterial‐feeding *Caenorhabditis elegans*, including its sensory system. The nervous system of *C. elegans* is simple and among the most well‐understood in animals; all neurons are identified, and the connectome is completely elucidated (Cook et al. 2019; White et al. 1986; Witvliet et al. 2021). The genetic tools and microsurgery system are well‐developed (Brenner 1974; Fang‐Yen et al. 2012), such that neurons and genes related to the nervous system can be manipulated. Second, although most nematode species have simple nervous systems similar to that of *C. elegans* (Schafer 2016), they have a variety of life histories including bacterial feeders, fungal feeders, plant parasites, animal parasites, and predators. Furthermore, the average nematode genome size ranges from ∼80 to 100 Mb (https://wormbase.org and https://parasite.wormbase.org/index.html), regardless of their diverse life histories (e.g., Blanc‐Mathieu et al. 2017; Eves‐van Den Akker et al. 2016; Kanzaki et al. 2018; Kikuchi et al. 2011). This relatively narrow range of genome sizes facilitates comparative genomics across different nematode species. Therefore, determining the genes, neurons, and neural systems that change concurrently with ecological factors is relatively simple.

Comparative morphological studies of the sensory system of bacterial‐feeding nematodes shed light on the morphological similarity of sensory neurons. For example, detailed observations of amphid neurons, the largest nematode sensory organ, showed that *C. elegans* has 12 amphid neurons (Ward et al. 1975). Here we categorize them into four types. The first has a single cilium (ASE, ASG, ASH, ASI, ASJ, and ASK neurons), and the second has double cilia (ADF and ADL neurons). The cilia of both types of neurons extend into a slit‐shaped pore and are exposed to the external environment. The third type has wing‐like cilia whose tips are buried internally (AWA, AWB, and AWC neurons). The fourth type has dendrites that are embedded within the sheath cell, with many villi. In addition, amphidal neurons of bacterial feeders including *Acrobeles complexus* and *Pristionchus pacificus* were categorized into one of the four types, although the number of each type of neuron differed (Bumbarger et al. 2009; Hong et al. 2019).

In plant‐parasitic nematodes of Tylenchoidea (e.g., *Meloidogyne* spp. and *Heterodera* spp.), amphid neurons are categorized into three types: the first type (single cilium), the second type (double cilia), and the fourth type (multiple villi). In addition to that, they appear to have amphid neurons that cannot be categorized into any of these four types (e.g., Endo 1980; Wergin and Endo 1976). These types of neurons were described as outer accessory cilia in previous studies. Endo (1980) reported that these neurons exhibit the characteristic morphology of amphid neurons, that is, forming an adherens junction among amphid neurons and entering a sensory channel formed by the amphidal sheath cell. Therefore, in this study, we consider outer accessory cilia to be amphid neurons and refer to these unique neurons as type V neurons tentatively. These types of sensory neurons are similar to AWA, AWB, and AWC neurons of *C. elegans* in that their terminals are highly branched and embedded within sheath cells. However, their cilia lie lateral and parallel to the amphidal canal, ascend toward the lateral cephalic sector and terminate beneath the hypodermis, which distinguishes their morphology from that of AWA, AWB, and AWC neurons. This classification suggests that plant‐parasitic nematodes evolved different sensory systems compared with bacterial‐feeding nematodes. However, the amphid neurons of plant‐parasitic nematodes have not been studied in detail or in a manner that permits direct comparison with the amphid neurons of *C. elegans*. For example, the composition of amphid neurons and the evolutionary origin of type V neurons remain unknown.

**FIGURE 1:**
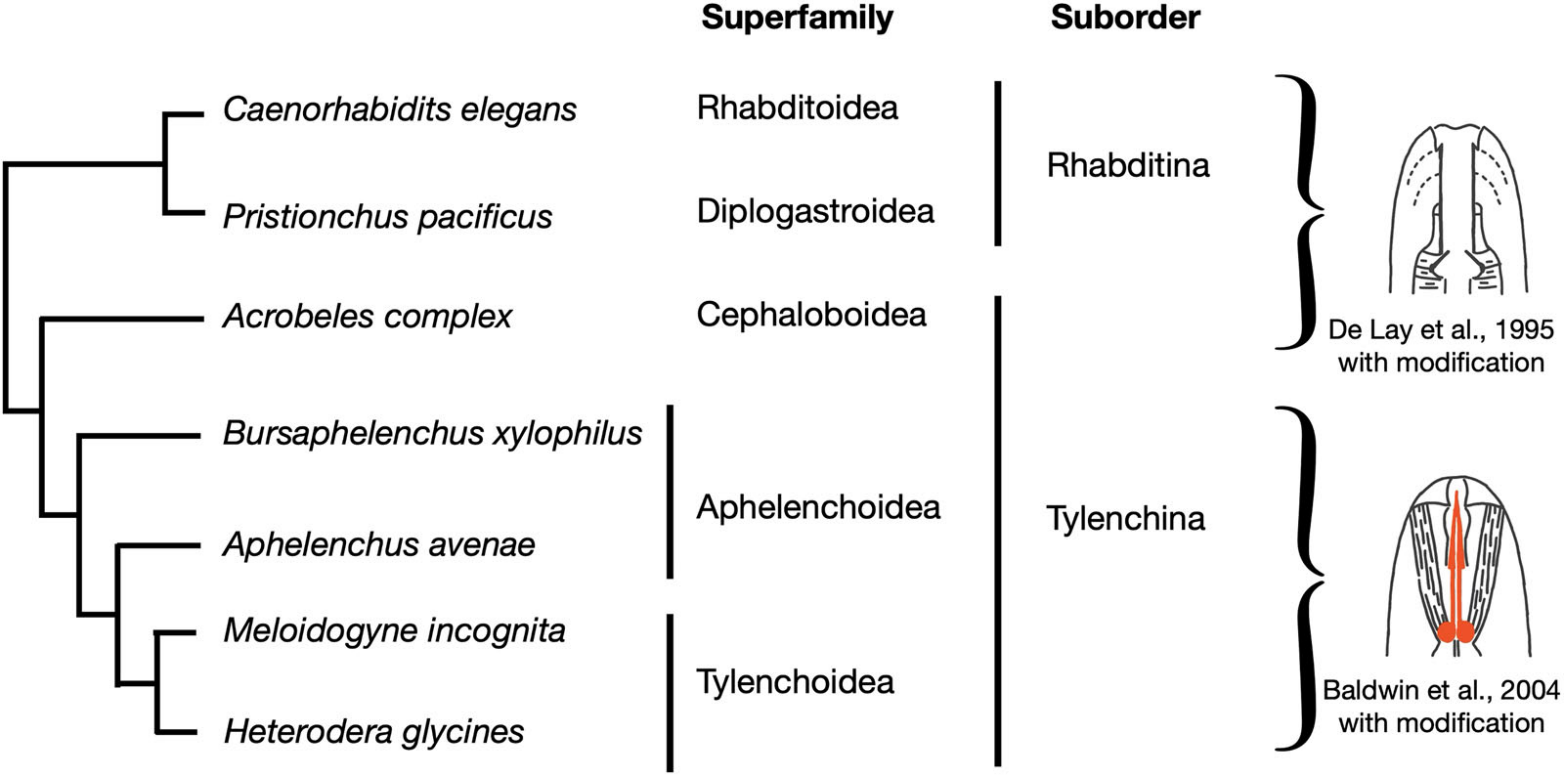
Phylogenetic relationships among Rhabditina and Tylenchina nematode species. Right images show the stoma morphology of each nematode group, indicating that Rhabditoidea, Diplogastroidea, and Cephaloboidea have cylindrical mouthparts and Aphelenchoidea and Tylenchoidea have stylets. Images were modified from De Ley et al. (1995) and Baldwin et al. (2004). Phylogenetic tree was constructed with reference to Qing et al. (2025).

In this study, we focused on a fungal‐feeding plant‐parasitic nematode of Aphelenchoidea, *Bursaphelenchus xylophilus*. In the natural environment, *B. xylophilus* inhabits the interior of pine trees, where it feeds on fungi growing in this environment. However, *B. xylophilus* also feeds on the parenchyma cells of pine trees and causes pine wilt disease. Therefore, this species is recognized as both a fungal‐feeding and plant‐parasitic nematode. Phylogenetically, *B. xylophilus* is positioned between plant‐parasitic nematodes of Tylenchoidea, such as *Meloidogyne* spp. and *Heterodera* spp., and bacterial feeders (Figure 1). Therefore, *B. xylophilus* is optimal for investigating the evolutionary origins of the type V neurons. To date, there have been no studies of the morphological characteristics of amphid neurons in *B. xylophilus*.

In this study, we investigated whether *B. xylophilus* possesses type V neurons similar to those described in other stylet‐bearing species, and comprehensively described the morphology of all amphid neurons using serial section transmission electron microscopy (TEM) and three‐dimensional (3D) reconstruction. A comparison of these features with those of *C. elegans* and other nematodes provided insight into the evolution of the nematode sensory system in relation to ecological and morphological innovations.

## 2 Materials and Methods

### 2.1 Nematode Preparation

*Bursaphelenchus xylophilus* Ka4 C1 (RRID: WB‐STRAIN:WBStrain00041429) was cultured at 25°C in 90‐mm Petri dishes containing fungal mats of the grey mold *Botrytis cinerea* on 1.5% malt extract agar medium (Difco, BD Biosciences, Franklin Lakes, NJ, USA) containing 4% agar and 100 µg chloramphenicol/mL. To collect nematodes in the adult female stage, nematode development was synchronized according to the methods of Shinya et al. (2009). To compare with the nervous system of the hermaphrodite of *C. elegans*, 1‐day‐old adult females were used for this study.

### 2.2 TEM Analysis

Females of *B. xylophilus* were frozen with a high‐pressure freezing apparatus (EM HPM 100, Leica, Wetzlar, Germany), and freeze substitution was performed in an auto‐freeze‐substitution apparatus (EM AFS2, Leica) with an acetone cocktail composed of 2% osmium tetroxide, 0.1% uranyl acetate, and 2% water (Mulcahy et al. 2018). During freeze substitution, nematodes were held at –90°C for 110 h, warmed to –20°C over a period of 5 h, held for 16 h, warmed again to 0°C over a period of 5 h, and then held at room temperature for 2 h. Specimens were then rinsed with pure acetone three times, and again after 1 h. Specimens were infiltrated with 25% Poly/Bed resin/acetone (Poly/Bed, Polysciences, Warrington, PA, USA) overnight, and infiltrated with 50%, 75%, 100%, 100%, and 100% resin/acetone following the method of McDonald (2014). Resin was polymerized at 60°C for 48 h.

Serial sections (50 nm) of the anterior parts of two adult females (2306C1 and 2306C2) were prepared using an ultramicrotome (UCT, Leica) fitted with a diamond knife (ultra 35°, Diatome, Bern, Switzerland). Sections were collected on formvar‐coated 2 mm × 0.5 mm SynapTek grids (DOT‐0.5, Nissin EM, Tokyo, Japan). One set of serial sections (2306C2) was stained with EM Stainer (Nissin EM) for 30 min followed by lead citrate (Sigma‐Aldrich, St. Louis, MO, USA) for 5 min using the Grid Staining Matrix System (Pelco, Fresno, CA, USA). Sections were imaged by TEM (JEM‐1400 Flash, JEOL, Tokyo, Japan) at 100 kV. Images of the anterior parts of the 2306C1 and 2306C2 samples were acquired in montages of nine individual images assembled automatically using a montage system. About 200 images of 2306C1 were aligned and 3D images of each neuron were constructed in 2306C1 using IMOD software (https://bio3d.colorado.edu/imod/, RRID:SCR_003297) (Kremer et al. 1996). Models were transferred to Blender v4.3.2 (https://www.blender.org/, RRID: SCR_008606) and modified for final visualization.

## 3 Results

**FIGURE 2:**
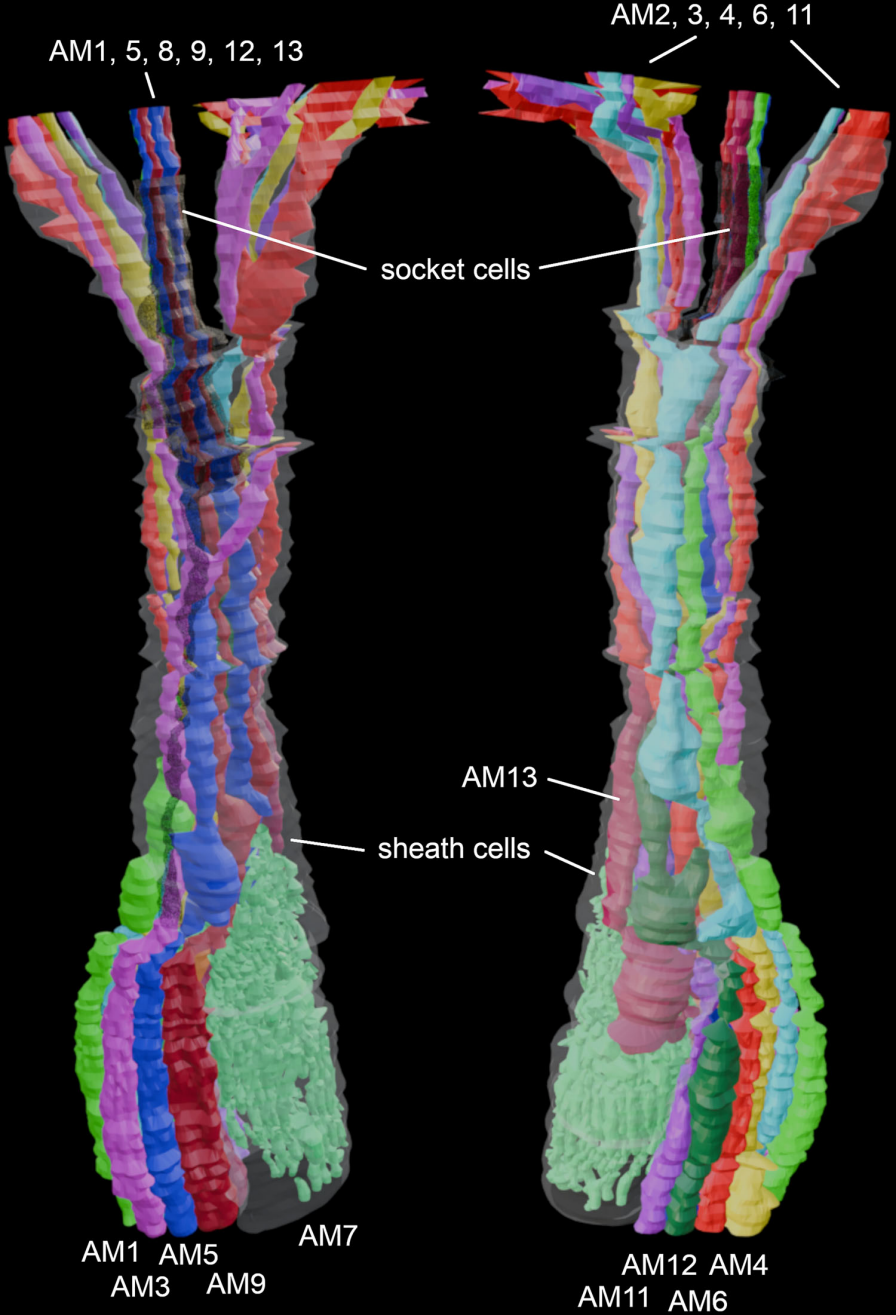
Three‐dimensional (3D) reconstructions of each amphid neurons, socket cell, and sheath cell of the anterior part of *Bursaphelenchus xylophilus*. The 3D image of the upper part was omitted because the cell membranes of the socket and sheath cells were not clearly distinguishable. The anterior amphid channels, which contain the dendrites of AM1, AM5, AM8, AM9, and AM12, and AM13 were surrounded by socket cells. At the same position, the dendrites of AM2, AM3, AM4, AM6, and AM11 existed in the sheath cells. AM7 was completely covered by sheath cell.

**FIGURE 3:**
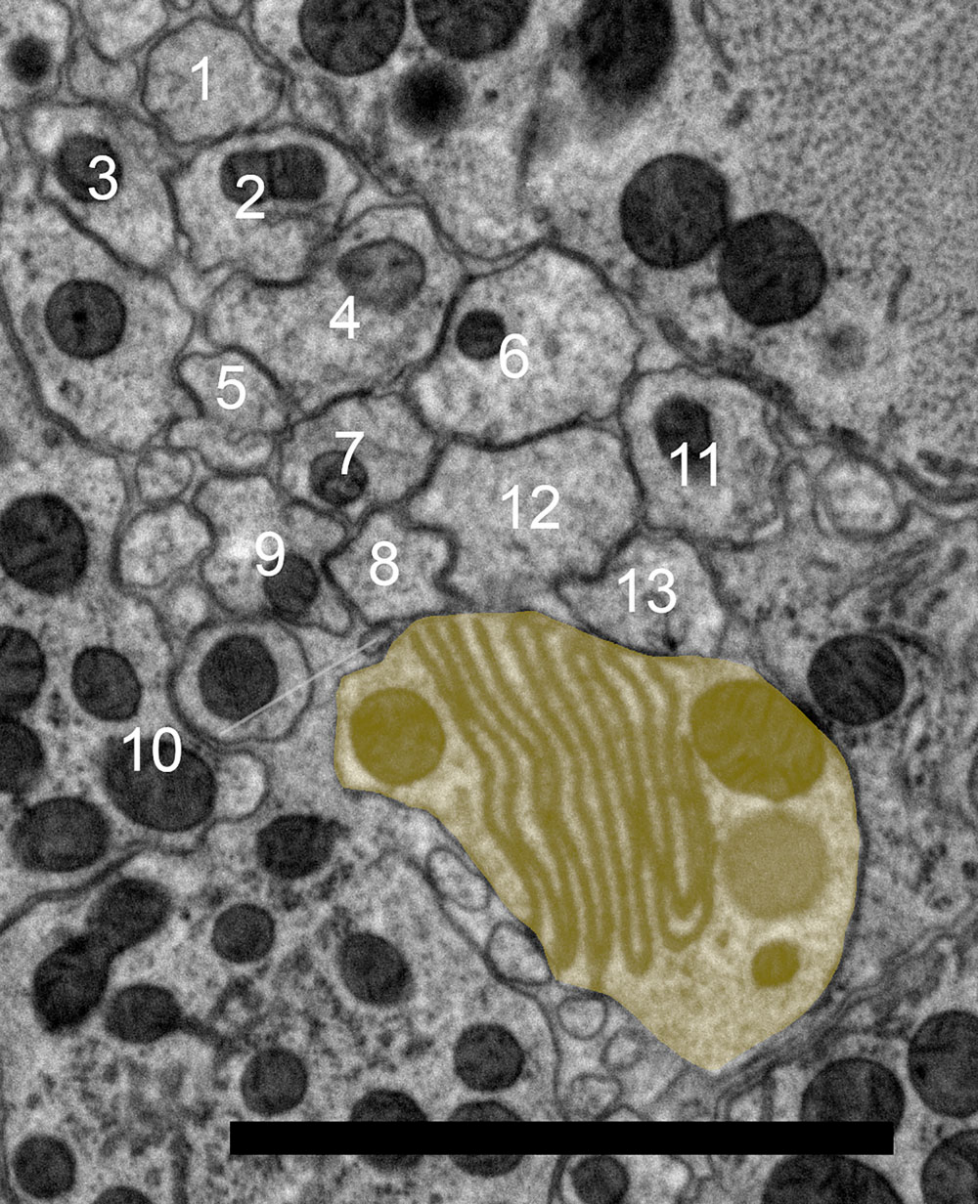
Transverse transmission electron microscopy (TEM) section of the anterior part of *Bursaphelenchus xylophilus* at a Golgi body of a presumed amphidal sheath cell. Numbers are the numbers of amphid neurons. Yellow shading indicates the amphidal sheath cell. The dorsal direction is oriented toward the top of the image. Scale bar = 2.0 µm.

**FIGURE 4:**
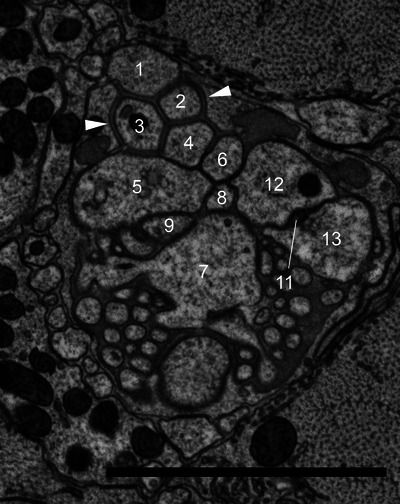
Transverse TEM section of the anterior part of *B. xylophilus* at a the adherens juction. Numbers are the numbers of amphid neurons. White arrows indicate adherens junctions between amphid neurons. The dorsal direction is oriented toward the top of the image. Scale bar = 2.0 µm.

The amphid of *B. xylophilus* consists of 13 sensory neurons (AM1‐13), 11 of which (except AM7 and AM10) enter a sensory channel formed by a sheath cell (Figure 2). The sheath cell has a Golgi apparatus distal to the posterior direction of the adherens junction (Figure 3) and in the vicinity of the axon of the amphid neurons. Mitochondria and transparent particles appeared in the sheath cell. Amphid neurons form an adherens junction where the dendrite enters a sensory channel (Figure 4). The anterior amphid channels, which contain the dendrites of AM1, AM5, AM8, AM9, sAM12, and AM13 (defined as type I and II neurons in the paragraph below), were surrounded by socket cells (Figure 2), which are connected by a self‐junction. At the same position, the dendrites of AM2, AM3, AM4, AM6, and AM11 (type V neurons) existed in the sheath cells (Figure 2).

### 3.1 Description of *B. xylophilus* Amphid Neurons

**FIGURE 5:**
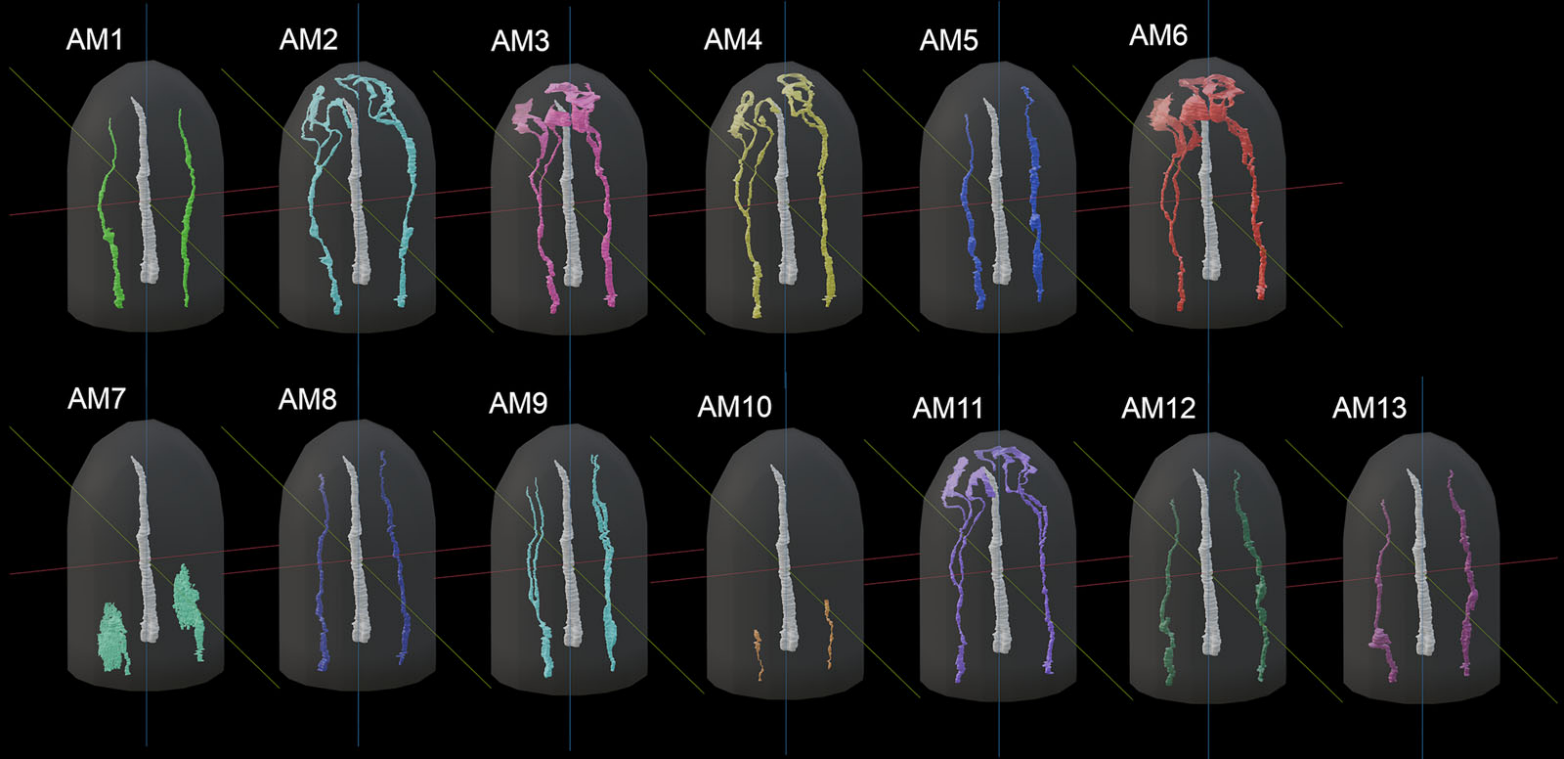
3D reconstructions of pairs of amphid neurons in *B. xylophilus*. Type I neurons including AM1, AM5, AM8, AM10, AM12, and AM13 Type I neurons had a single cilium connected to the amphidial pore. except AM10. AM10 formed no cilium and did not enter the amphid channel formed by socket cells. Type II neurons including AM9. Type II neurons had double cilia connected to the amphidial pore. Type III neurons including AM7. Type III neurons had a cilium and had microvilli below the adherens junction. Type V neurons including AM2, AM3, AM4, AM6, and AM11. The cilia of type V neurons are divided into three branches proximal to the anterior direction of the cephalic framework. The stylet was represented as a white object. The dorsal side is shown at the front right, and the ventral side at the back left.

**FIGURE 6:**
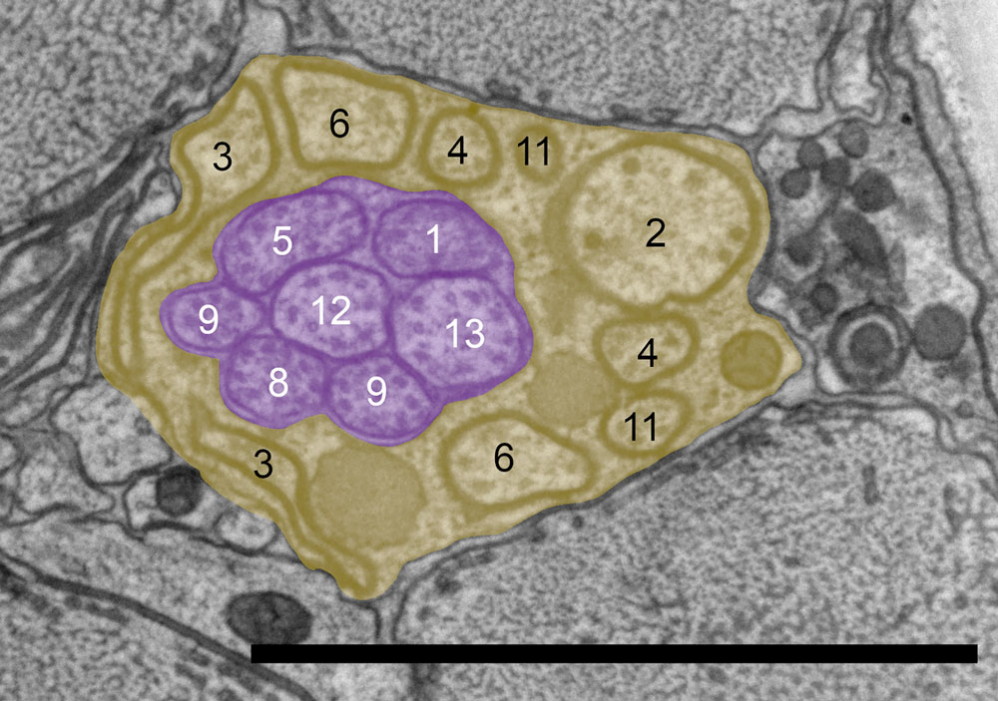
Transverse TEM image of the anterior part of *B. xylophilus* at the location of an amphidial pore. Numbers are the numbers of amphid neurons. The dorsal direction is oriented toward the top of the image. Red and yellow shading indicate an amphidial pore and a presumed amphidal sheath cell. Scale bar = 2.0 µm.

**FIGURE 7:**
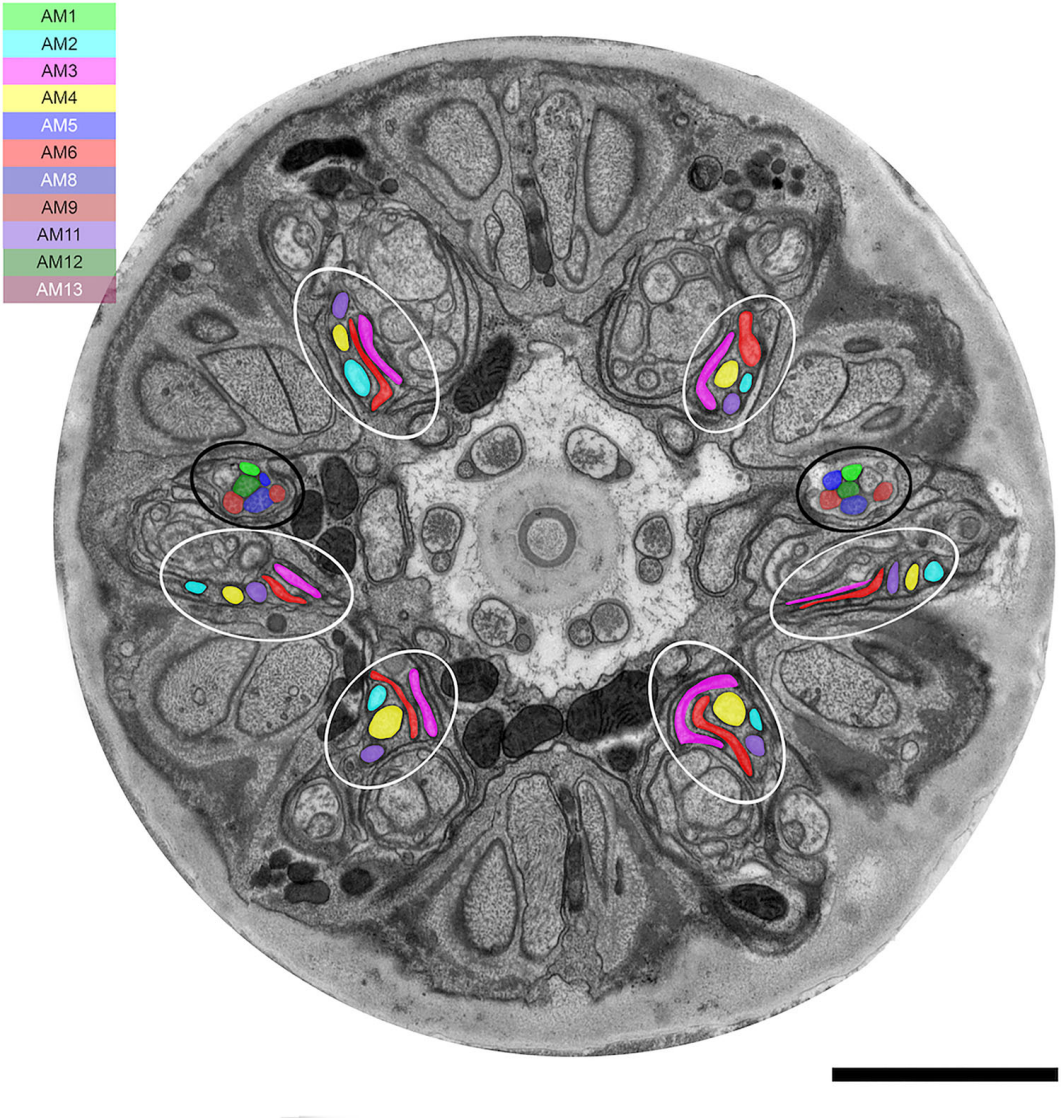
Transverse TEM whole image of the anterior part of *B. xylophilus* proximal to the anterior direction of cephalic framework. Cilia of type I and II neurons (AM1, AM5, AM8, AM9, AM12, and AM13) are indicated by black circles. Cilia of type V neurons (AM2, AM3, AM4, AM6, and AM11) are divided into three (white circles). The dorsal direction is oriented toward the top of the image. Scale bar = 2.0 µm.

**FIGURE 8:**
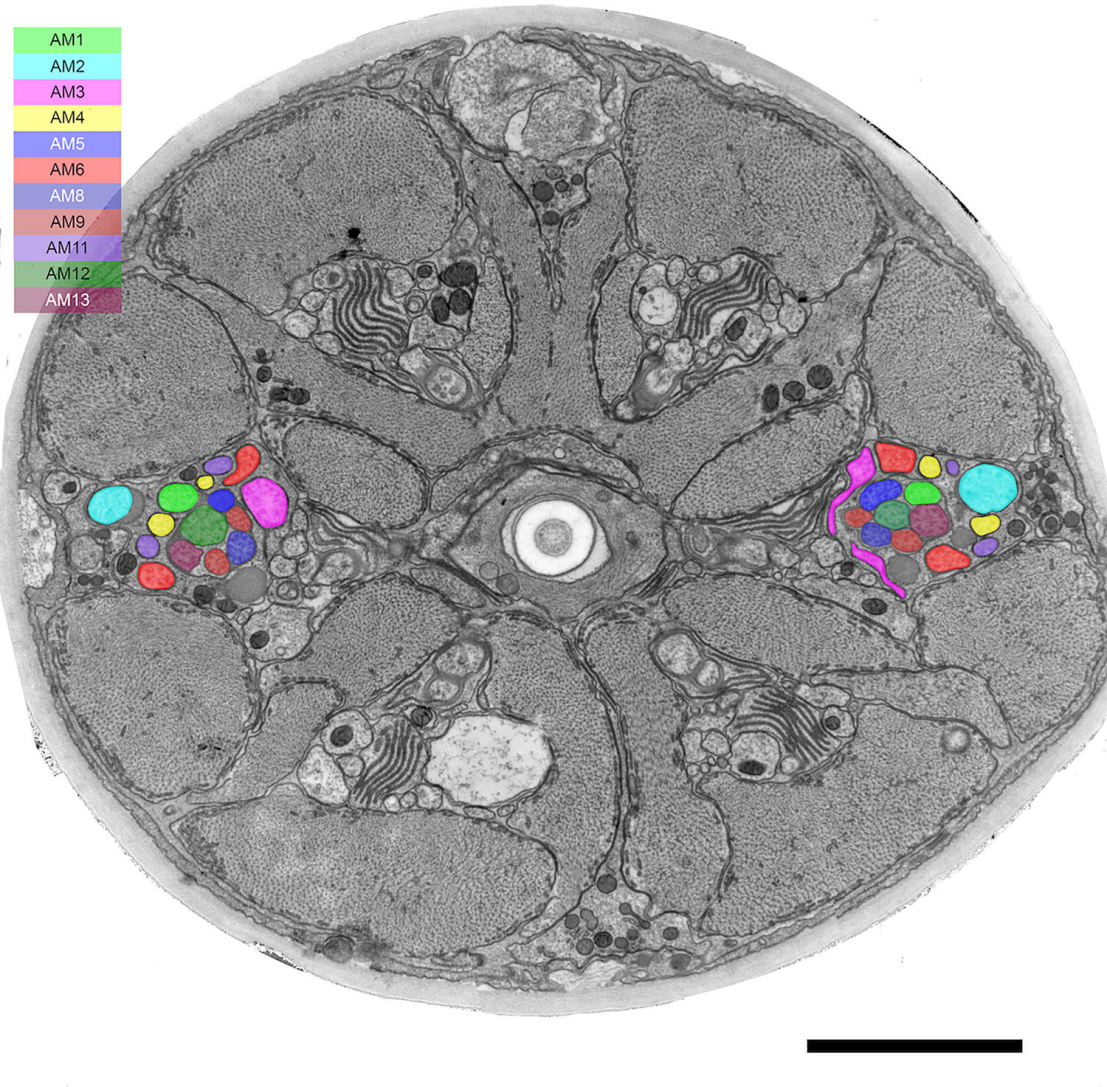
Transverse TEM whole image of the anterior part of *B. xylophilus* proximal to the posterior direction of the cephalic framework. Cilia of a type V neuron (AM2) were fused into one, and the others (AM3, AM4, AM6, and AM11) were divided into two in this area. The dorsal direction is oriented toward the top of the image. Scale bar = 2.0 µm.

We identified 13 amphid neurons (AM1–AM13) and classified them into four morphological types (Table 1, Figure 5). In this study, the classification was made solely on the basis of ultrastructural features. Neurons with wing‐like cilia, corresponding to type III in *C. elegans*, were not observed in *B. xylophilus*. Six neurons (AM1, AM5, AM8, AM10, AM12, and AM13) were categorized as type I (Figure 5), having a single cilium connecting to the amphidial pore (Figure 2) except AM10. The cilia of type I neurons were proximal to the anterior direction of the adherens junction (Figure 5). Although AM10 was exceptional, its neurite formed adherens junctions with other neurites and entered the sensory channel, but it did not form a cilium or extend into the amphid channel formed by socket cells. One neuron (AM9) was categorized as type II (Figure 5), having double cilia connecting to the amphidial pore (Figure 5). The neurite was divided into two proximal to the anterior direction of the adherens junction. The cilia of type II neurons were proximal to the anterior direction of the adherens junction. No neuron was categorized as type III, having wing‐like dendrites. One neuron (AM7) was categorized as type IV (Figure 5), having a cilium and microvilli below the adherens junction (Figure 4). The microvilli invaginate the adjacent sheath cell. The cilia of type IV neurons were proximal to the posterior direction of the adherens junction. Five neurons (AM2, AM3, AM4, AM6, and AM11) were categorized as type V (Figure 5), having cilia that were not connected to the amphidial pore, but occurring within the sheath cell (Figure 2). The cilia divided into three branches proximal to the anterior direction of the cephalic framework (Figure 5, 6, 7). The divided cilium connected different lips (Figures 5, 6, 7). The cilia of type V neurons were proximal to the anterior direction of the adherens junction. The arrangement of amphid dendrites at the posterior entrance into the sheath cell is shown in Figure 8.

**TABLE 1:** Categorization of amphidal sensory neurons of female *B. xylophilus*.

	Description	Composition	Presumably homologous amphid neurons of *C. elegans*
Type I	A single cilium is connected to the amphid pore	AM1, AM5, AM8, AM10, AM12, and AM13	ASE, ASG, ASH, ASI, ASJ, and ASK
Type II	Double cilia are connected to the amphid pore	AM9	ADF and ADL
Type III	Wing‐like cilia exist in sheath cell	—	AWA, AWB, and AWC
Type IV	No cilia are present, but microvilli exist below the adherens junction	AM7	AFD
Type V	Cilia are not connected to the amphid pore but exist in the sheath cell	AM2, AM3, AM4, AM6, and AM11	No

## 4 Discussion

### 4.1 Comparison of Amphid Neurons Between *B. xylophilus*, *C. elegans*, and Other Nematodes

**FIGURE 9:**
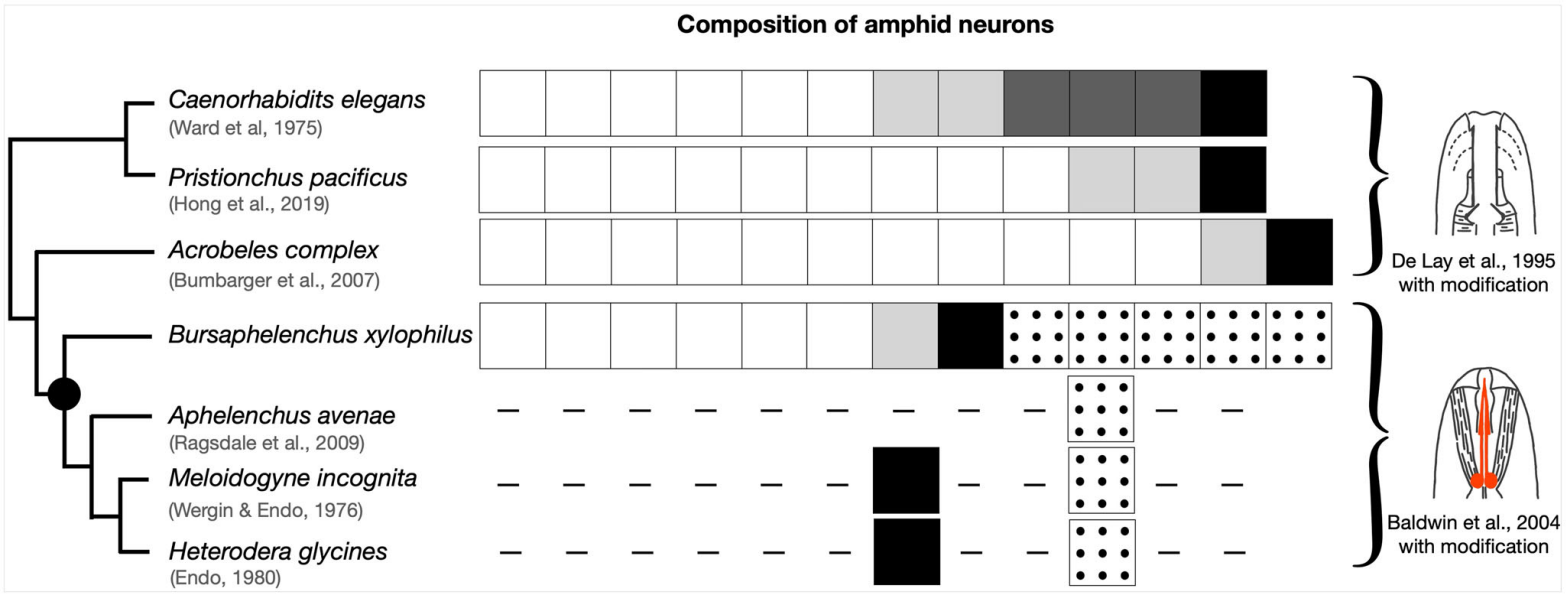
Comparison of amphid neuron compositions among nematode species based on a phylogenetic tree. White boxes indicate type I neurons homologous with the ASE, ASG, ASH, ASI, ASJ, and ASK neurons of *Caenorhabditis elegans*. Light gray boxes indicate type II neurons homologous with ADF and ADL neurons of *C. elegans*. Dark gray boxes indicate type III neurons possessing wing‐like cilia; that is, AWA, AWB, and AWC neurons of *C*. *elegans*. Black boxes indicate type IV neurons homologous with AFD neurons. Dotted boxes indicate type V neurons homologous with outer accessory cilia of plant‐parasitic nematodes of Tylenchoidea. Dashed lines indicate that no data are available. Right images show the stoma morphology of each nematode group, indicating that Rhabditoidea, Diplogastroidea, and Cephaloboidea have cylindrical mouthparts, whereas Aphelenchoidea and Tylenchoidea have stylets. Black dot indicates the tentative time at which type V neurons and stylets evolved concurrently. Images were modified based on Baldwin et al. (2004) and De Ley et al. (1995). Phylogenetic tree was constructed with reference to Qing et al. (2025).

The morphologies of type I neurons except AM10 of *B. xylophilus* were essentially the same as those of ASE, ASG, ASH, ASI, ASJ, and ASK neurons in *C. elegans*, type II neurons corresponded to ADF and ADL, and type IV neurons resembled AFD. AM10 shows strong similarity to the ASA neuron in *Acrobeles complexus*. Type I, II, and IV neurons are present in bacterial feeders such as *C. elegans*, *Pristionchus pacificus*, and *A. complexus*, while some animal‐parasitic Tylenchina species (e.g., *Parastronglyoides trichosuri*, *Strongyloides stercoralis*) lack type II neurons (Ashton et al. 1995; Zhu et al. 2011). Previous studies described type IV neurons in the plant‐parasitic *Meloidogyne incognita* and *Heterodera glycines* (Endo 1980; Endo and Wergin 1977). Although type I–II neurons were also reported, their distinction was not clearly described. Therefore, we refrain from discussing type I–II neurons in these species (Figure 9). Similarly, TEM images of *Aphelenchus avenae* show cilia that could correspond to type I–II neurons (Ragsdale et al. 2008, 2009), but we do not include them here for the same reason.

The morphology of type V neurons is similar to that of outer accessory cilia in *H. glycines* (Endo 1980) and *M. incognita* (Endo and Wergin 1977). Because these sensory neurons formed adherens junctions with other amphid neurons, we categorized these neurons as amphid neurons. We also concluded that *A. avenae* had type V neurons based on TEM images from previous studies (Ragsdale et al. 2008; see Figure 5C, Ragsdale et al. 2009; see Figure 7F). Although these authors did not refer to amphid neurons, structures characteristic of type V neurons were observed in TEM images of *A. avenae*. Some cilia of *A. avenae* were not connected to the amphidial pore, but were presumably present in the amphid sheath cell, near the anterior part of cephalic framework. No previous studies have reported type V neurons in bacterial feeders.

### 4.2 Evolutionary Origin and Phylogenetic Distribution of Type V Neurons

We identified unique structured amphid neurons in the pine wood nematode *B. xylophilus*. These type V neurons share a striking morphological similarity with the outer accessory cilia previously described in plant‐parasitic nematodes such as *Meloidogyne* spp. and *Heterodera* spp. (Endo 1980; Wergin and Endo 1976). Notably, these neurons are absent in bacterivorous nematodes, suggesting a taxon‐specific trait associated with Tylenchida.

*Bursaphelenchus xylophilus* belongs to the superfamily Aphelenchoidea, which is phylogenetically situated between the bacterivorous Cephaloboidea and the plant‐parasitic Tylenchoidea. Importantly, no type‐V‐like neurons have been observed in *A. complexus* (Cephaloboidea), supporting the hypothesis that these neurons evolved in the common ancestor of *Meloidogyne*, *Heterodera*, and *Bursaphelenchus*. Alternatively, type V neurons may be broadly associated with parasitic lifestyles, as type V neurons (or outer accessory cilia) have been observed only in parasitic taxa. However, TEM images of the non‐parasitic fungal feeder *A. avenae* (Ragsdale et al. 2009) also appear to show structures similar to type V neurons, suggesting that type V neurons are not exclusive to plant parasites.

**FIGURE 10:**
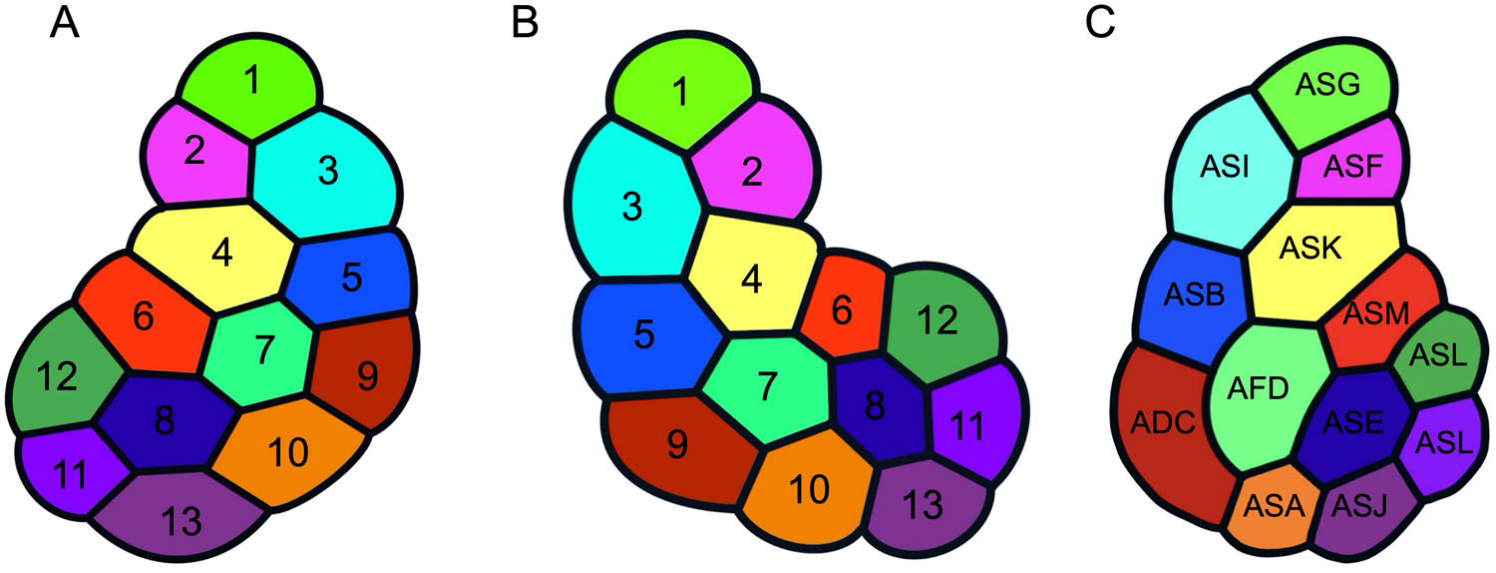
Arrangement of amphid dendrites at the entrance into the sheath cell of *B. xylophilus* (A and B) and 1st stage juvenile of *Acrobeles complexus* (C). The top side of each image are dorsal. The right side of image A and left sides of images B and C are adjacent to the pharynx. The cells represented in the same color are considered homologous between *B. xylophilus* and *A. complexus* based on their position at the entrance into the sheath cell. There were no positional differences between 2306C1 and 2306C2 samples.

The arrangement of amphid dendrites at the posterior entrance into the sheath cell provides important information for discussing homologous amphid neurons among species (Figure 10). For example, the positions of the type II and IV neuron dendrites are approximately the same between *B. xylophilus* and the first juvenile stage of *A. complexus*, the closest relatives of *B. xylophilus* for which data are available. It suggests that type II and IV neurons are homologous in these two species. Furthermore, the overall arrangement at the posterior entrance into the sheath cell in *B. xylophilus* is similar to that of *A. complexus*. This suggests that, beyond the type II and IV neurons, sensory neurons occupying equivalent positions across species may also be homologous. In the case of type V neurons, their positions correspond to those of type I neurons in *A. complexus*. Specifically, the positions of AM2, AM3, AM4, AM6, and AM11 match those of ASF, ASI, ASK, ASM, and ASJ, respectively. In *H. contortus*, the ASC neuron in the first‐stage juvenile becomes the AWC neuron in the infective third stage, indicating that type I neurons can be morphologically variable (Li et al. 2000; 2001). Based on this, one hypothesis is that type V neurons evolved from the type I neurons of bacterivorous nematodes. Another hypothesis is type V neurons are homologous to AWA, AWB, and AWC neurons of *C. elegans* because there is morphological similarity between the two; their terminals are highly branched and embedded within sheath cells. To determine which hypothesis is correct, future studies will need to examine the cell body's distribution and perform functional analyses.

The presence of a stylet, a cellular piercing apparatus, was well correlated with the distribution of type V neurons. All nematodes known to possess these neurons also possess a stylet, and bacterivorous species lacking a stylet also lack type V neurons. Thus, type V neurons and the stylet may have co‐evolved. Stylets have also evolved independently in Dorylaimida and Triplonchida. In *Xiphinema americanum* (Dorylaimida), cephalic sensory structures have been described (Wright & Carter 1980), although no clear type V neurons were described. Interestingly, the internal sense organs of *X. americanum* bear a partial morphological resemblance to type V neurons, in characters such as dendritic tips located beneath the lip cuticle and the lack of connection to the amphidial pore. Functional analyses of these structures in both Tylenchida and Dorylaimida are required to determine whether they share evolutionary or functional homology.

### 4.3 Diversity in Amphid Neuron Composition Among Nematodes

Across nematodes within Rhabditina and Tylenchina, the total number of amphid neurons is relatively conserved, typically ranging from 11 to 13 (e.g., Ward et al. 1975; Bumbarger et al. 2009; Hong et al. 2019). However, the composition of amphid neurons in *B. xylophilus* differs significantly from that of bacterivorous species such as *C. elegans*.

In *B. xylophilus*, a total of five type V neurons were identified. By contrast, neurons with wing‐like cilia (AWA, AWB, and AWC), which are involved in olfaction, were absent. Additionally, one type II neuron was missing compared with *C. elegans*. Given that II neurons are associated with gustation (Bargmann et al. 1990) and type III neurons mediate volatile odor detection (Bargmann et al. 1993; Troemel et al. 1997), these differences suggest that *B. xylophilus* has a distinct chemosensory profile that is potentially reduced in scope compared with those of bacterivorous nematodes.

These structural differences may reflect adaptations to ecological niches. *C. elegans* is isolated from diverse environments such as urban gardens, compost, fruit, and riverbanks and is exposed to a broad range of sensory cues (Frézal and Félix 2015). Conversely, *B. xylophilus* is consistently associated with pine trees, where the variety of sensory cues derived from hosts and predators is comparatively limited. Therefore, reduced chemosensory modalities may be sufficient in this ecological context.

### 4.4 Functional Hypotheses Regarding Type V Neurons

Morphologically, type V neurons are distinguished by trifurcated cilia, each extending toward a distinct lip region without connecting to the amphidial pore. This unique arrangement implies a specialized function distinct from other amphid neurons. Endo and Wergin (1977) and Wergin and Endo (1976) proposed that this morphology reflects a mechanosensory role.

Supporting this view, *A. avenae* (Aphelenchoidea) exhibits stylet‐thrusting behavior only after pressing its lips against a substrate at right angles to the longitudinal axis (Fisher & Evans 1967), suggesting that mechanical contact is a behavioral cue for feeding initiation. Bacterivorous nematodes lack a stylet, instead ingesting bacteria via cylindrical mouthparts, and show no such behavior, that is, exhibit feeding behavior only after pressing its lips against a substrate at right angles to the longitudinal axis. Therefore, the distribution and behavior of stylet‐equipped nematodes support the hypothesis that type V neurons act as lip‐based mechanoreceptors, detecting mechanical stimuli that trigger stylet deployment.

Nevertheless, morphological similarity does not necessarily equate to identical function. Despite the lack of type III neurons, *B. xylophilus* responds to volatile cues from pine trees, insect vectors (Zhao et al. 2007), and sex pheromones (Shinya et al. 2015). Given their branched morphology reminiscent of AWA, AWB, and AWC neurons in *C. elegans*, type V neurons may also contribute to olfactory functions. However, this remains speculative in the absence of direct functional evidence.

## 5 Conclusion

Our study provides the first detailed 3D reconstruction of amphid neurons in *B. xylophilus* and identifies type V neurons as a potential evolutionary innovation linked to stylet‐bearing nematodes. Although their morphology suggests mechanosensory roles, their precise function remains to be determined.

We are currently pursuing the identification of type V neuron cell bodies and their functional ablation using laser microsurgery, a well‐established technique in *C. elegans* neurobiology (Fang‐Yen et al. 2012). This approach will allow us to directly test the behavioral consequences of type V neuron loss, further elucidating their role in environmental sensing and nematode evolution.

## Data Availability

The data that support the findings of this study are available from the corresponding author upon reasonable request.
